# Reducing impact load on RC-Slabs using Expanded Polystyrene (EPS)

**DOI:** 10.1038/s41598-025-04903-7

**Published:** 2025-06-20

**Authors:** Yosra El-Maghraby, John Wael, Aya Assem, Ahmad E. Khalil

**Affiliations:** Faculty of Engineering, British University in Egypt, Cairo, 11837 Egypt

**Keywords:** Reinforced concrete (RC) slabs, Expanded polystyrene foam (EPS), Impact force dissipation, Reducing impact load, Experimental testing, Civil engineering, Mechanical engineering

## Abstract

Reinforced Concrete (RC) slabs are widely used in structural applications due to their ability to withstand heavy loads. However, under impact loading conditions such as falling objects or debris, their brittle nature makes them prone to cracking and damage. To address this, Expanded Polystyrene (EPS) has been explored as an energy-absorbing material capable of reducing the severity of impact forces. Traditionally used as an insulating material, EPS possesses favorable mechanical properties—lightweight, high deformability, and cushioning capacity—that have led to its application in civil infrastructure as geofoam and lightweight fill. Despite its growing use, the potential of EPS as a protective surface layer for RC slabs under impact loading remains underexplored. This study investigates the effectiveness of a surface-mounted EPS layer in reducing the impact response of RC slabs. Six full-scale RC slab specimens were tested under vertical impact from a 90 kg steel ball dropped from a height of 1 m. Half of the specimens were cast as control slabs, while the other half included a 5 cm thick EPS layer atop the concrete. Accelerometers were used to capture dynamic responses, and a detailed finite element model was developed in ABAQUS, incorporating experimentally measured material properties and contact interaction at the EPS–concrete interface. The model accounted for separation and frictional behavior between the two materials. Experimental and numerical results showed that the EPS layer significantly reduced the maximum acceleration, displacement, and energy dissipation within the concrete slab compared to the control specimens. While control slabs absorbed more energy through cracking and damage, the EPS slabs exhibited reduced structural deterioration, indicating more efficient impact mitigation. These findings highlight the potential of EPS as a cost-effective solution to enhance the impact resistance of RC slabs. Future work will focus on parametric studies involving EPS thickness, EPS density, steel reinforcement ratio, intensity of impact load and concrete material properties to generalize the results for broader applications.

## Introduction

An unexpected, dynamic force delivered rapidly to a structure or system is known as an impact load. It represents an unexpected change in both direction and size that generates a dynamic force. Impact loads can be caused by a variety of events, including collisions, explosions, and abrupt energy releases. A static load, which is a continuous force exerted over an extended length of time, is different from an impact load. Impact loads include things like a vehicle accident, a hammer striking a nail, and rockfalls large objects crashing onto a surface^1^.

These days, concrete construction and well-designed concrete structures are not enough to overcome the difficulties of the modern world. Even when static loads are expected to happen well, sudden dynamic loads such as wind with powerful hooks, impact knockouts, explosions, rockets, and missiles cause an enormous impact on the structure that might result in a sudden and unexpected collapse. The structure’s dynamic behavior, which is affected by impact loads, is extremely complicated and directly connected to the kind of construction that was designed as well as the differences in the properties of each material. High strain rates and a sudden increase in energy brought on by dynamic loading may be too much for the structure to manage in a short amount of time. Capacity is what determines the mechanical and physical reaction. The structure’s capacity to absorb and release energy into the surrounding environment quickly is what determines the physical and mechanical reaction^2^.

Expanded polystyrene (EPS) has seen an exponential increase in interest in the building industry in recent years. EPS is made up of tiny polystyrene beads that are produced by polymerizing styrene. Expanded polystyrene (EPS) is a well-known insulating material that finds usage in various applications because of its significant properties such as light weight, rigidity, excellent thermal insulation, and significant impact resistance. Furthermore, it offers longer service life, little maintenance needs, quick and affordable construction, complete water and vapour barrier, insulation for regulated temperatures, and a high load-bearing capacity at a light weight. Formed and consisted of tiny, spherically shaped particles that are around 98% air, EPS foam is a thin, lightweight cellular foam. The microcellular closed cell structure of EPS is the source of its strong insulation and shock-absorbing properties^3^. In addition, EPS can be mixed with the concrete mixture (Cement, Sand, Aggregate) as a fourth parameter^4^. This mixture would enhance the concrete durability, but would decrease the compressive strength, and this parameter will be discussed in the paper.

Expanded polystyrene (EPS) panels are light in weight and consist of closed-cell structures. Produced by compressing polystyrene beads with steam and a blowing agent, EPS panels feature numerous small air chambers and come in various sizes, thicknesses, and densities to meet different insulation needs^3^. EPS is commonly used to insulate outer walls, roofs, and floors, EPS panels act as thermal barriers to prevent heat transfer and reduce sound transmission. Their lightweight and thin design makes them easy to install and handle, as they can be cut to fit specific spaces. EPS layers can also be mixed into concrete for efficient insulation in building envelopes. Additionally, EPS panels have anti-mold and anti-mildew properties, making them suitable for various construction applications. They can be recycled and repurposed at the end of their life cycle. It is crucial to follow the manufacturer’s installation and maintenance guidelines to ensure their effectiveness and safety^3^.

The use of an EPS layer above reinforced concrete (RC) slabs is grounded in both practical applications and research-based systems. In particular, Insulated Concrete Form (ICF) systems widely utilize EPS panels as permanent formwork and insulation, demonstrating the feasibility of placing EPS externally on concrete elements in real-world construction. This configuration has gained popularity in both structural and energy-efficient building applications due to its thermal insulation, reduced construction time, and improved impact resistance^2^.

One of the most frequent geological risks, particularly in mountainous regions, is rockfalls. Roadways and other infrastructures are particularly at risk of rockfalls due to their powerful dynamic properties and unpredictability. Therefore, engineers try to make new progress in prevention against the rockfall disaster in the recent years^5^. There are both active and passive ways to counteract rockfall, which could prevent the risk of happening or protect structures that are exposed to it^6^. Sheds, retaining walls, and flexible nets are examples of passive protection techniques. The metal flexible net, which consumes the most energy in the flexible protection system, is braided using unique steel wires that have a solid surface and high strength^7^. For creating rock-sheds, frames with a padded slab on top are most frequently utilized. Consequently, the rock-shed structure and the cushioning materials compose the two components of the impact-resistant system of a rock-shed^8^.

It is necessary to integrate energy-consuming parts or cushion layers to the side of the structure that is in contact with the falls to prevent fractures and reduce the impact of rockfalls on flexible sheds or retaining walls^5^. The cushion layer seeks to effectively distribute impact force and absorb energy to lessen the impact load on the RC frame. Increasing the thickness of the soil cushion enhances the impact resistance of the structure by providing additional energy absorption. However, it also increases the deadload, which can affect structural performance and lead to higher construction costs due to the need for larger soil volumes and associated design adjustments. However, tiny cushion layers are insufficient to provide adequate protection from rock-sheds or to reduce impact loads. As a result, the limitation mentioned above directly limits the use of cushion layers. Foam’s low density and affordable price make it a preferred material to improve cushioning performance. Foam material dissipates impact energy, cushioning the slab below^8^. Now, the most suitable method for dissipating the energy of rockfalls is to use a cushion layer. The cushion layer can reduce the maximum impact force of a rockfall by increasing the duration of contact, lowering the rockfall’s acceleration, and distributing the contact force over a broader area. This mitigates the associated risks from rockfall particles. Therefore, an effective impact-resistant system relies primarily on two key components: the structural concrete elements (such as slabs and columns) and the cushioning materials designed to absorb and dissipate impact energy^5^.

A field-scale rockfall impact test was conducted to evaluate the performance of reinforced concrete (RC) slabs placed on sandy soil cushions of varying depths^5^. The test involved dropping weighted rocks from different heights to analyze the effects on acceleration duration, mid-point displacement, and strain in the concrete slab. The results indicated that the thickness of the cushion layer has an exponential relationship with the applied impact force. Using an empirical formula, the maximum penetration closely matched the test outcomes. Notably, a cushion layer thickness of 0.6 m reduced the impact force to approximately 70% of the maximum force. The study concluded that increasing the cushion layer thickness exponentially dampens the impact force. The thickness of the cushion layer significantly affects the impact force, suggesting that enhancing this thickness can optimize impact force reduction. This is particularly relevant for surfaces with cushion layers, where the impact force of rockfalls has an exponential relationship with the layer’s thickness. The energy dissipation effect of the cushion layer is advantageous, as demonstrated by the strain distribution, with the bottom rebar layer experiencing higher strain values than the top layer. The RC slab undergoes several stages under cumulative impact energy: initial bending crack formation, propagation of additional bending fractures, initiation of shear cracks, and progression to higher bending crack penetration leading to failure. The study also reviewed RC slab failure mechanisms and mitigation methods.

An experimental and numerical investigation was conducted to analyze the response and failure mechanisms of simply supported two-way reinforced concrete (RC) slabs under low-velocity impact forces^9^. Nine RC slabs were tested experimentally, focusing on impact energy and reinforcement ratios. Impact loads were applied using drop-weight test equipment at varying heights. Measurements included accelerations, collision loads, and displacements over time. Results showed that increasing the rebar ratio enhanced the slabs’ bending strength, stiffness, and toughness, reducing maximum and residual displacements but increasing accelerations. Higher rebar ratios also improved the slabs’ load-carrying capacity. Conversely, lower rebar ratios and higher impact energy led to more damage and wider cracks. Finite element analysis, used for validation, successfully predicted maximum acceleration, displacement, and impact loads but showed stiffer behavior due to the homogeneous concrete representation. Numerical analysis revealed faster vibration damping compared to experimental results. The correlation between numerical and experimental data supports using the finite element model to evaluate dynamic responses and failure mechanisms of RC slabs under low-velocity impacts. This model is reliable for assessing critical responses like maximum acceleration, impact load, and displacements during the design phase^9^.

Furthermore, an experimental study was conducted to evaluate the ability of rock-sheds to withstand rockfall impacts by proposing a new cushion layer above the rock-shed slabs. This cushion consists of two layers: soil and expanded polyethylene (EPE). This design aims to reduce deadweight, enhance impact resistance, and lower maintenance costs compared to traditional designs. Laboratory uniaxial compression tests were conducted on EPE and expanded polystyrene (EPS) specimens to assess their compression performance. Variables for EPS included thickness, loading rate, and density, with density affecting durability while thickness and loading rate did not. EPE’s durability was unaffected by these variables and exhibited minimal residual deformation upon compression. Due to its superior resilience, EPE was chosen for the cushion layer. Significant rockfall impact studies on reinforced concrete (RC) rock-sheds were conducted to evaluate the efficiency of various cushion layers. Under low impact energy, the sand cushion absorbed all the impact energy with minimal rock reaction. The sand-EPE layer exhibited higher kinetic energy dissipation than the sand-EPS layer. The sand-EPS layer demonstrated greater dissipation capacity, whereas sand-EPE showed better cushioning performance. Subsequent tests with higher impact energy revealed that the sand-EPE layer was preferred, with the structure sustaining damage at 108.4 kJ impact energy and collapsing at an eight-meter drop height. Failure mechanisms for EPE included bending and punching shear failures. In conclusion, the EPE layer provided superior impact resistance and substructure protection, making it the preferred choice for the new cushion layer in rock-sheds^8^.

Moreover, several studies have explored EPS as an impact-mitigating layer. For example, El-Yilmaz et al.^5^ demonstrated that RC slabs covered with EPS on the impact side, experienced reduced damage and enhanced energy absorption under drop-weight impact loads. Similarly, Cao et al.^6^ investigated hybrid slabs combining RC, Ultra-High-Performance Concrete (UHPC), and EPS, and reported improved flexural behavior and synergy between materials. These findings confirm the validity and relevance of adopting an EPS-topped RC slab configuration in both experimental studies and practical applications.

Additionally, a large-scale blast test on concrete slabs was conducted to evaluate the performance of various protective treatments under blast loading^10^. Sixteen slabs were tested: eight with five different protective techniques and eight as control specimens without reinforcement. The tests examined two distances: at 0.5 m, the explosion caused significant local damage that completely penetrated the slabs. In this scenario, the reinforcements did not provide additional protection or help retain fragments. At one meter, the slabs exhibited fewer deflections and were not fully penetrated. In these tests, reinforcements helped retain some fragments and reduced permanent deflections. The study revealed that determining whether the protective treatments enhanced the slabs’ resistance was challenging. However, a few key observations emerged. Carbon fiber mesh reinforcement showed desirable deflection differences compared to non-reinforced slabs, suggesting it warrants further investigation. Adding steel and polypropylene fibers improved the slabs’ mechanical properties, particularly tensile strength, which should be assessed at various scaled distances for close-in explosions. On the other hand, using a steel plate as reinforcement caused more damage than leaving the slab unreinforced. In conclusion, the effectiveness of different protective treatments on concrete slabs under blast loading remains unclear based on the measured criteria for evaluating explosion damage. Further research is needed to clarify these findings^10^.

Moreover, an experimental and numerical investigation conducted to evaluate and analyze the metal flexible net’s impact resistance against falling rocks was examined. Experiments were conducted on the metal flexible net’s resistance to diagonal impacts. The experiments related finite element models (FEM) were established. Three features were compared between the results of the experiments and the FE model: the maximum instantaneous impact force carried by the supporting rope, the metal flexible net’s deformation properties and the impact force’s time-history curve as expressed by the supporting rope. The FE model’s validity is confirmed. A strong FE analysis platform is developed to investigate metal flexible nets’ ability to withstand impacts from falling rocks. When the metal flexible net is properly inclined, it could impact rock falls out of safe areas, prevent rock falls from staying on the net, and perform self-cleaning tasks. Fourteen-face precast polyhedron concrete blocks with shapes compliant with SAEFL criteria were used in the test rock fall described in the research. The forms of falls of rock vary in nature. In terms of the flexible protection system, the metal flexible net’s damping and cushioning become more noticeable as rock edges and corners collapse. The construction can efficiently withstand the impact of falling boulders, as demonstrated by the experiment’s results, and it can be widely and commercially implemented along rock slopes and roadways^7^.

Impact force is a critical consideration in engineering applications, particularly for reinforced concrete slabs subjected to rockfalls on buildings and roads. This research focuses on using Expanded Polystyrene (EPS) as a cushion material above the concrete layer to mitigate impact damage. The study aims to reduce both the damage from rockfalls and the associated maintenance costs for reinforced concrete slabs. The primary purpose of using Expanded Polystyrene (EPS) as a cushioning material in the RC slab impact load study is to investigate its effectiveness in mitigating the impact forces transferred to the structural element. EPS, due to its lightweight, energy-absorbing properties, and favorable compressive behavior, serves as a buffer layer that can dissipate kinetic energy during impact events. By incorporating EPS between the impacting object and the RC slab, the study aims to assess its potential in reducing damage to the slab, prolonging structural integrity, and enhancing overall impact resistance. This approach is particularly relevant in scenarios where structures are exposed to rockfall or similar dynamic loading conditions. This application is supported by the study conducted by El Maghraby and Metwally (2019), which demonstrated that EPS panels effectively absorb a portion of the impact energy when applied to RC slabs. Their experimental results indicated that slabs incorporating EPS panels could withstand approximately double the impact energy compared to control specimens without EPS, highlighting the material’s efficacy in impact mitigation^2^.

An ABAQUS CAE^11^ model analysis was carried out to investigate the properties of EPS on the reinforced concrete slab. The results of the model were compared with the experimental test performed at the lab. For this experiment, spherical rockfall models with different masses were built, along with a rockfall impact test. Test results are presented in this study, including the RC slab’s strain response at multiple measuring points, the rockfall’s acceleration duration curve with varying mass and impact height, the dynamic load’s duration curve on the RC slab’s surface, and the slab’s midpoint displacement after impact. To find the ideal cushion layer thickness, a relationship between the impact force on the cushion layer surface and the thickness of the layer was first developed using acceleration data. Second, a dimensionless factor was suggested to characterize the impact force transmission law in the cushion layer quantitatively based on the experimental data. Finally, an analysis of the dynamic failure modes and the process of failure of the RC slabs under different impact situations was carried out. Based on the above, the experimental results of the current study can serve as a basis for enhancing buildings that avert rockfall accidents.

## Experimental program

### Test setup

**Fig. 1:**
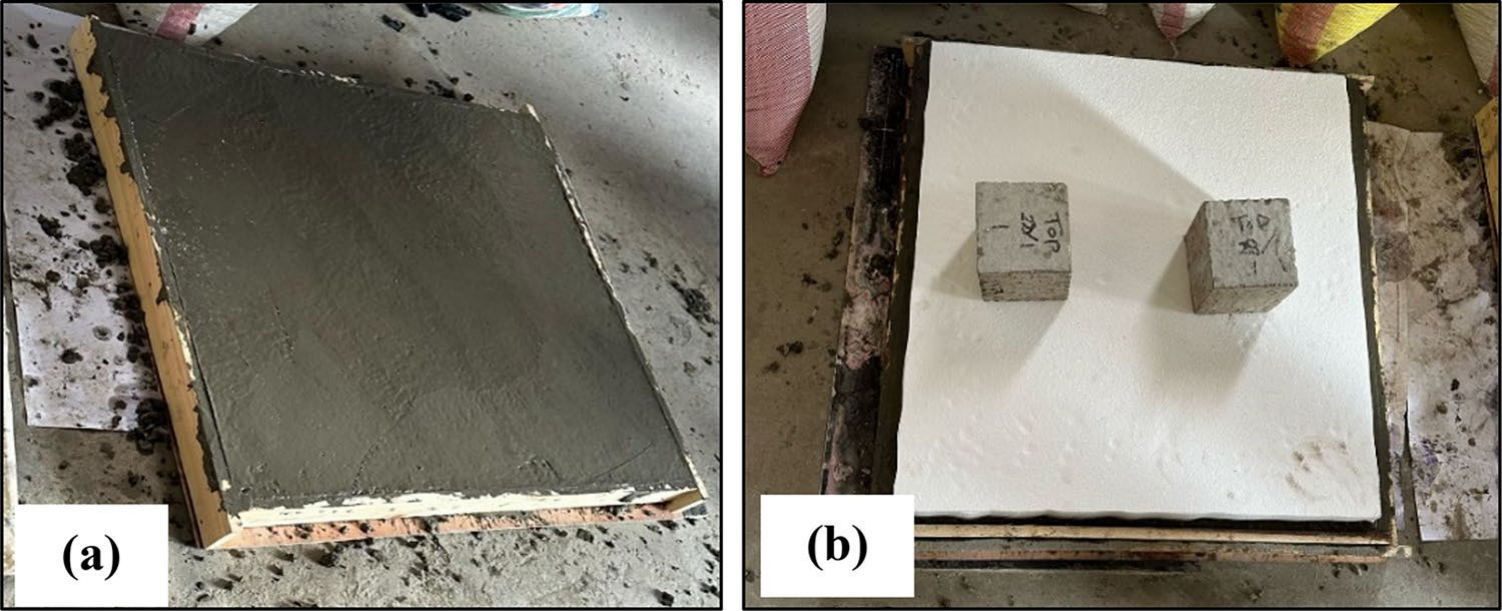
Reinforced concrete slab **(a)** Control Specimen and **(b)** EPS specimen.

**Fig. 2:**
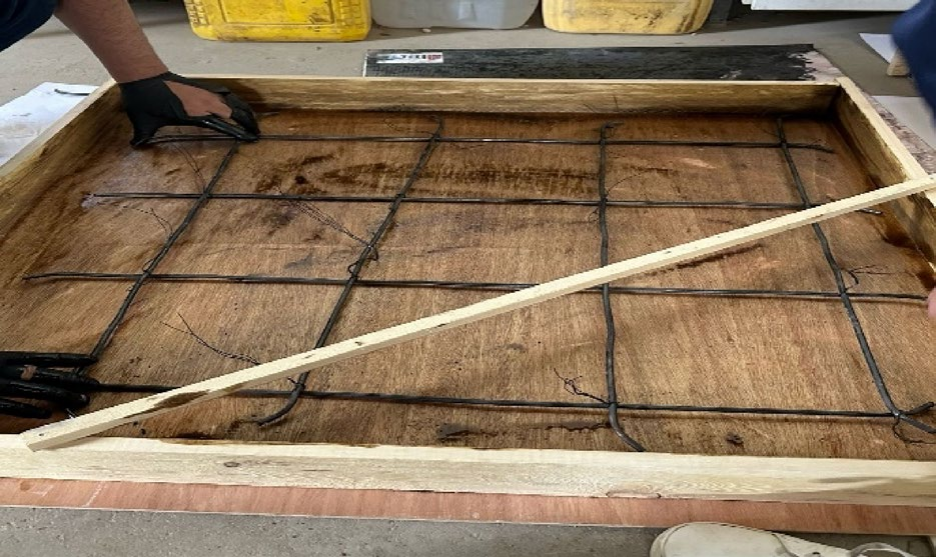
Reinforcement of concrete slabs.

**Fig. 3:**
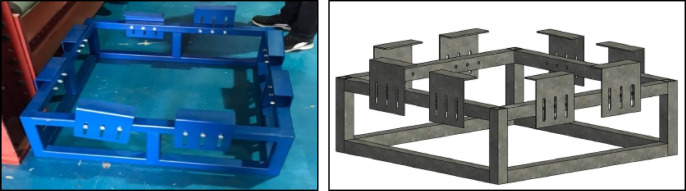
High strength steel impact table.

**Fig. 4:**
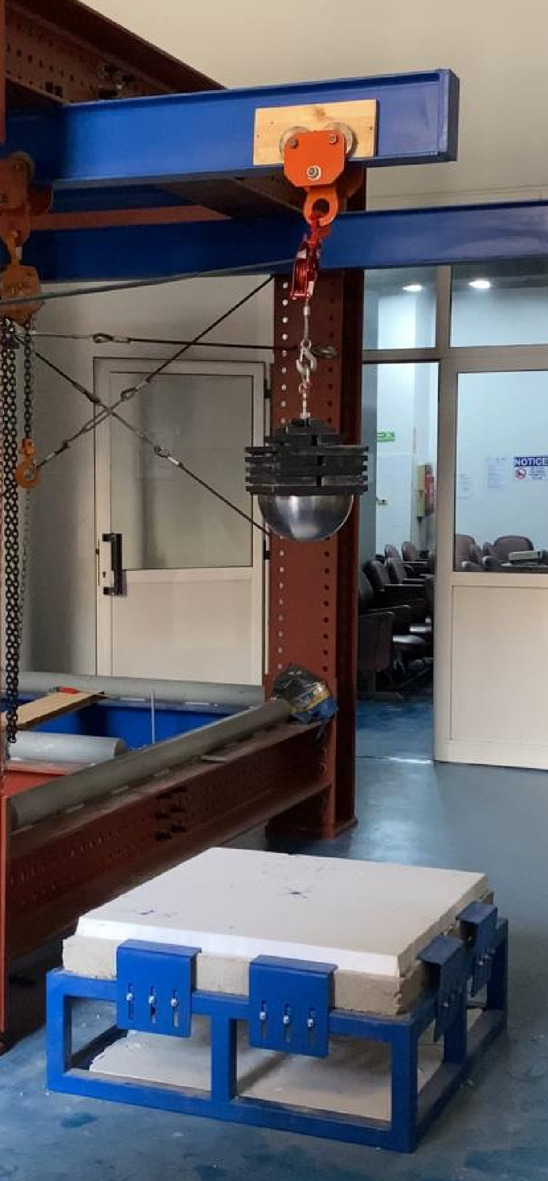
Test setup for impact load test (white EPS layer placed on the top of the RC slab).

Six experimental tests were conducted to evaluate the performance of Reinforced Concrete slabs under impact loading. Three of these tests were utilized as control specimens (without EPS), while the other three incorporated an Expanded Polystyrene (EPS) layer placed on top of the slab, acting as a cushion layer as shown in Fig. 1. The dimensions of the reinforced concrete slabs of the current test program are 90 cm x90 cm and a depth of 8 cm. Each slab was reinforced with mild steel reinforcement (240/360), featuring 4 bars of 8 mm diameter per meter run, resulting in a total of 4 bars in each direction within the slab as illustrated in Fig. 2. The experimental setup was carefully designed to simulate impact loading on RC slabs with fully fixed boundary conditions. A rigid steel reaction table was fabricated, incorporating four vertical steel supports firmly bolted to the base frame (Fig. 3). This allowed the test slabs to be clamped along all four edges, effectively simulating a slab that is fixed on all sides and preventing boundary displacement. To generate the impact load, a 90 kg solid steel ball was dropped from a height of 1 m, producing an impact energy of approximately 882 J. Each specimen was subjected to a single impact drop to ensure consistency and to capture the immediate response to impact load^12^. This setup enabled a controlled and consistent simulation of impact conditions across all specimens. The response was captured using accelerometers and a high-speed data acquisition system Fig. 4.

To capture the acceleration induced by the impact load, two accelerometers were installed on the underside of each slab specimen. An accelerometer is a device designed to measure dynamic responses such as vibration and acceleration within a structure. They operate based on inertial principles, typically employing the piezoelectric effect: an internal mass applies force to a piezoelectric element during motion, generating an electrical signal proportional to the acceleration experienced. Since the mass remains constant, the resulting output is directly related to the magnitude of acceleration.

The electrical signals produced by the accelerometers were conditioned and digitized using a data acquisition system, providing a time-history of acceleration with high temporal resolution. These acceleration-time records were subsequently processed through numerical integration techniques to derive the corresponding velocity and displacement responses of the slab under impact loading.

**Fig. 5:**
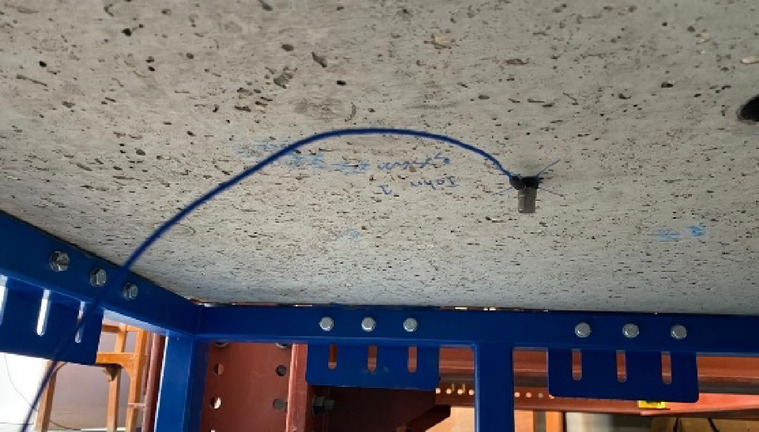
accelerometer placed at bottom of slab.

The accelerometers were fixed at the bottom of the slab but not centered to avoid potential damage from slab failure. Instead, two accelerometers were positioned each at 30 cm from the opposite edges, to ensure accurate measurements while minimizing the risk of damage as shown in Fig. 5. The acceleration data collected from the accelerometers was integrated over time to calculate the velocity of the slabs. By integrating the velocity data, the displacement of the slabs under impact loading was determined. This process of double integration allows for a comprehensive analysis of the dynamic response of the slabs, including the impact-induced accelerations, velocities, and displacements.

### Material properties

#### Concrete properties

In this study, the reinforced concrete slabs were constructed using a concrete mixture designed in accordance with Egyptian Code of Practice (ECP) for optimal workability, strength, and durability^13^. The concrete mix consisted of 328 kg of cement per cubic meter (kg/m³), 190 kg/m³ of water, a water-to-cement ratio of 0.58, 765 kg/m³ of fine aggregate, and 1148 kg/m³ of coarse aggregate. The selection of these materials and their proportions was based on standard practices to ensure that the concrete mix meets the necessary structural performance criteria^14^.

The compressive strength of the concrete was tested using cubes cast from the same mixture and cured for 28 days. The compressive strength values for the cubes corresponding to each of the six slabs were 25.85, 27.33, 24.52, 26.59, 28.48, and 29.91 MPa. These values showed some variability, which is expected due to inherent differences in material properties and curing conditions. The average compressive strength of these six slabs was calculated to be 27.11 MPa, providing a reliable measure of the concrete’s ability to withstand compressive loads.

**Fig. 6:**
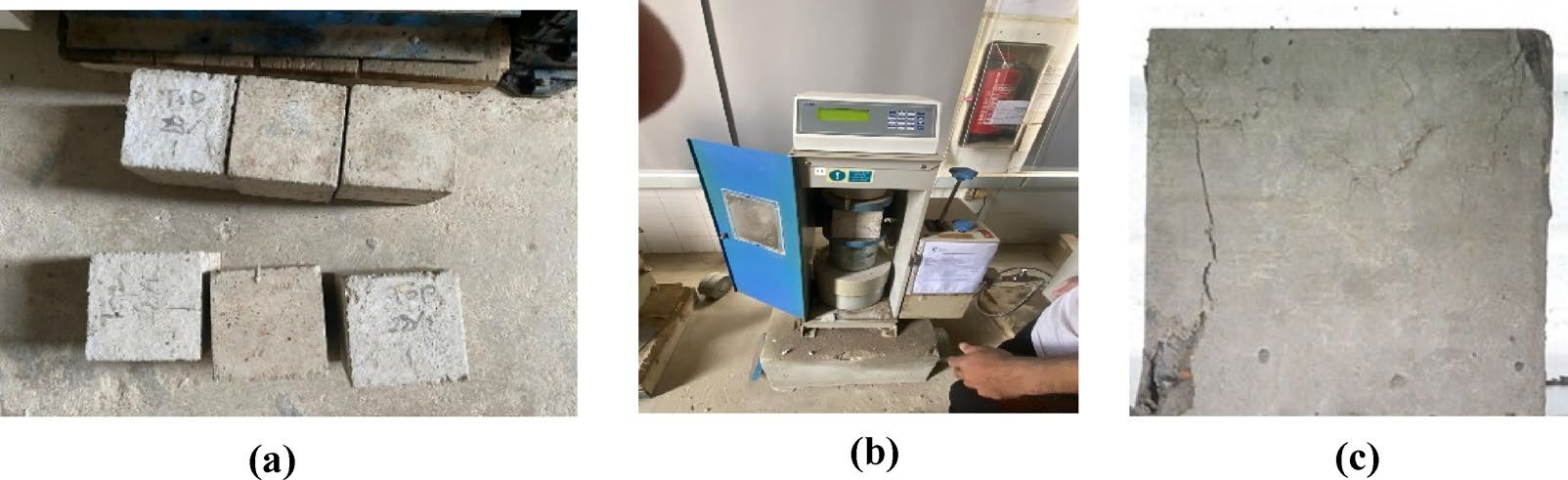
Concrete properties test **(a)** concrete cubes, **(b)** Compressive strength testing, and **(c)** Failure of cubes.

In summary, the concrete mixture used in this study was carefully designed and tested to ensure it met the necessary performance criteria for experimental investigation. The average compressive strength of 27.11 MPa indicates that the concrete is suitable for the intended application, providing a robust foundation for the subsequent impact load tests and analysis Fig. 6.

#### Steel reinforcement

In the reinforced concrete slab impact test, mild steel reinforcement was used. The mild steel employed in this study has a yield strength of 240 MPa, which is the stress level at which the steel begins to deform plastically. Before this point, the steel deforms elastically and will return to its original shape once the applied stress is removed. The ultimate strength of the mild steel, which is the maximum stress it can withstand while being stretched or pulled before breaking, is 360 MPa. This tensile strength indicates the maximum load the steel can handle before failure. Additionally, the diameter of the mild steel reinforcement used in the slab is 6 mm. The designed reinforcement significantly affects the load-bearing capacity and overall stiffness of the reinforced concrete slab and is selected based on design requirements. The combination of these properties ensures that the mild steel reinforcement provides adequate strength and ductility, enabling the reinforced concrete slab to absorb and dissipate the energy from impact loads effectively.

#### Expanded polystyrene (EPS)

The Expanded Polystyrene (EPS) used in this study has a thickness of 5 cm. EPS is a polymer primarily composed of polystyrene (C_8_H_8_)_n_, with a polystyrene content ranging from 95 to 100%. The material properties of the EPS layer are detailed in Table 1. These properties include its density, compressive strength, tensile strength, bending strength, and thermal conductivity. The EPS layer was selected for its excellent cushioning properties and its ability to absorb impact energy, which is crucial for protecting the reinforced concrete slabs under impact loading. Its lightweight nature and high compressive strength make it an ideal material for use in structural applications where impact resistance is required. The high polystyrene content ensures consistent performance and durability, maintaining its structural integrity under various loading conditions. The detailed material properties of the EPS layer provide a comprehensive understanding of its performance characteristics, which are essential for evaluating its role in the experimental tests. Table 2 summarizes the tested specimens.

**Table 1:** Material properties for EPS.

Properties	Test method	Value	Unit
Density		20	kg/m^3^
Compressive strength	EN 826	120	kPa
Tensile strength	EN 1607	113	kPa
Bending Strength	EN 12,089	162	kPa
Thermal Conductivity	EN 12,667	0.035	W/m K

**Table 2:** Tested Specimens.

Specimen	Dimensions (cm)	Fcu (Mpa)	Steel reinforcement		EPS Thickness (cm)	EPS Density (kg/m3)	Addition of EPS panel
			Diameter	Fy/Fu (Mpa)			
S1-1	90 × 90 × 8	25.85	6	240/360	N/A	N/A	No
S1-2	90 × 90 × 9	27.33	6	240/361	N/A	N/A	No
S1-3	90 × 90 × 10	24.52	6	240/362	N/A	N/A	No
S2-1	90 × 90 × 11	26.59	6	240/363	5	20	Yes
S2-2	90 × 90 × 12	28.48	6	240/364	5	20	Yes
S2-3	90 × 90 × 13	29.91	6	240/365	5	20	Yes

### Experimental results

**Fig. 7:**
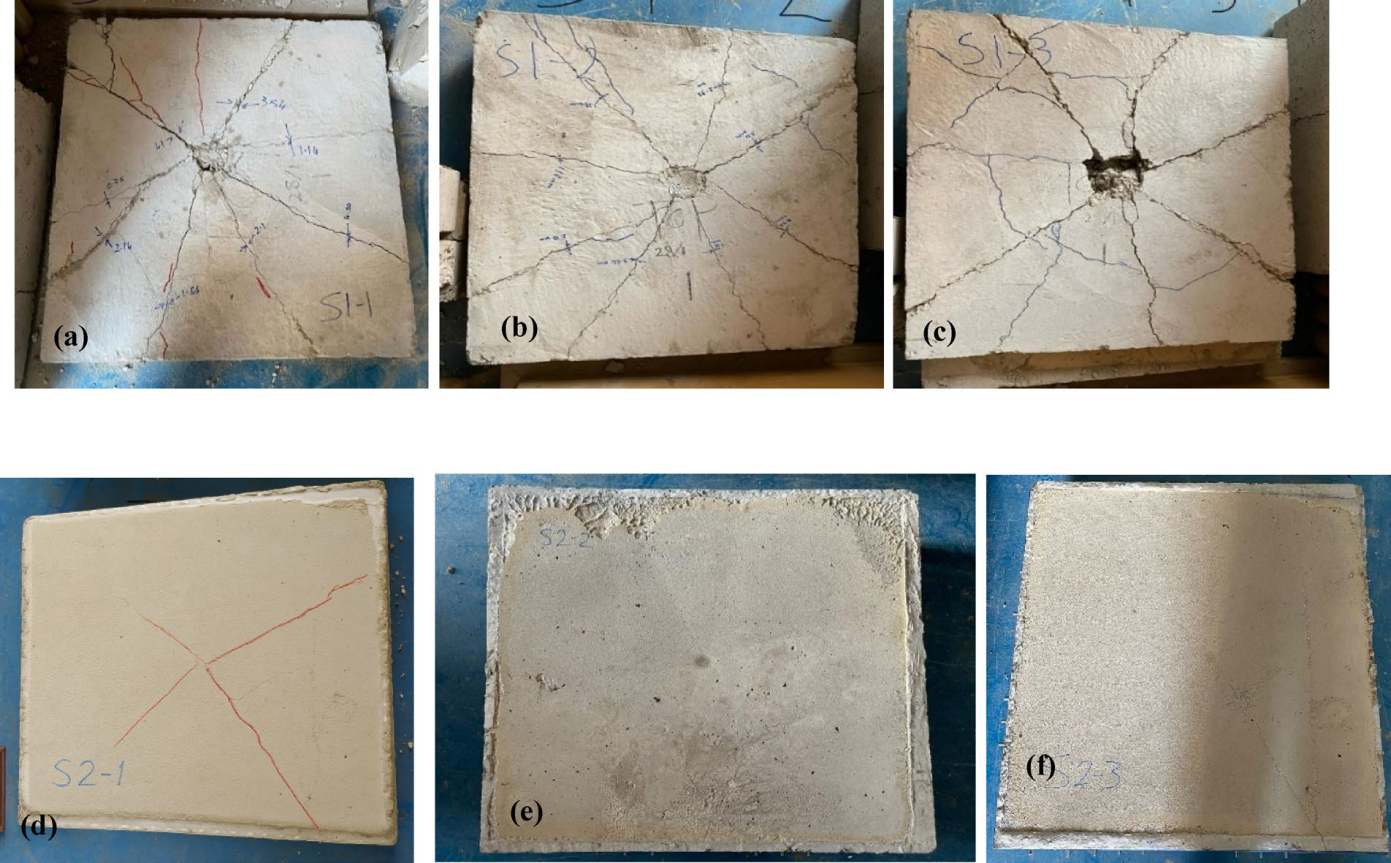
Top cracks in slab **(a)** S1-1, **(b)** S1-2, **(c)** S1-3, **(d)** S2-1, **(e)** S2-2, and **(f)** S2-3.

**Fig. 8:**
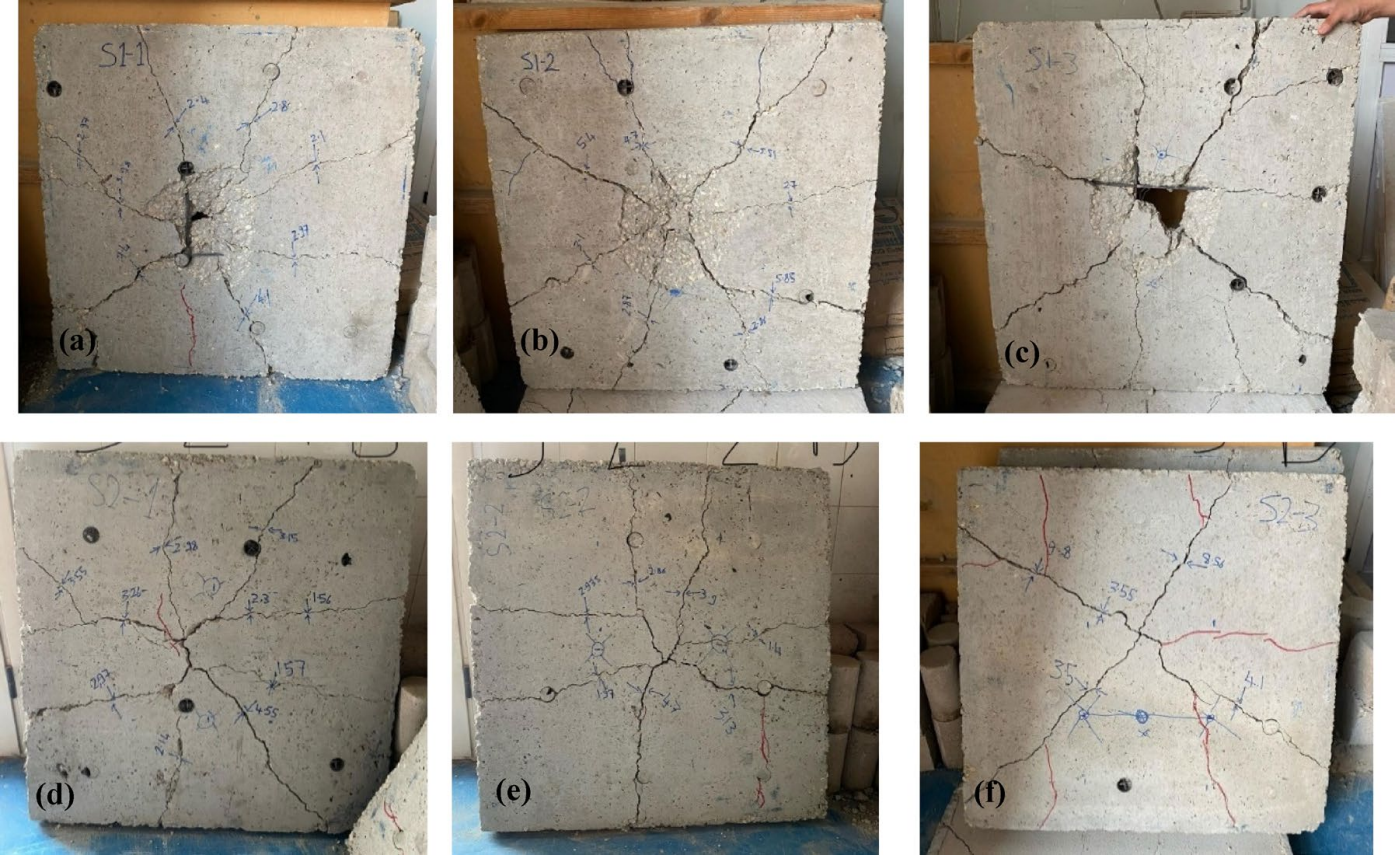
Bottom cracks in slab **(a)** S1-1, **(b)** S1-2, **(c)** S1-3, **(d)** S2-1, **(e)** S2-2, and **(f)** S2-3.

This section describes the results of the impact load experimental tests. The three control specimens are labeled as S1, and the three EPS specimens are labeled as S2. After the impact load was applied, the cracks that appeared in all specimens were measured at both the top and the bottom of the slabs. Figures 7 and 8 illustrate the cracks of all specimens. Additionally, the cracks were measured at eight points on both the top and bottom of the slabs, as shown in Tables 3 and 4, respectively.

The impact load test results reveal distinct differences in performance between the control slabs (S1) and the EPS slabs (S2). For the control slabs, significant top surface cracks were observed, with crack widths ranging from 0.14 mm to 7.55 mm, indicating substantial damage due to the impact load. In contrast, the EPS slabs displayed only hairline cracks on the top surface, suggesting superior resistance to surface cracking under impact loads. On the bottom surface, the control slabs exhibited considerable cracks, with widths reaching up to 9.55 mm, highlighting severe structural damage. The EPS slabs also experienced bottom surface cracks, but these were generally smaller and less severe than those in the control slabs. Notably, one EPS slab (S2-3) showed a crack width of 9.8 mm, indicating some variability in performance.

Regarding data variability, a statistical analysis was conducted on crack width measurements for both the control and EPS slab groups. For the control slabs (S1 group), two sets of data were analyzed. The top surface crack widths (22 values) yielded a mean ($\overline{x}$) of 2.77 mm, a standard deviation *(S.D)* of 1.53 mm, and a margin of error *(E)* of ± 0.68 mm, resulting in a 95% confidence interval between 2.10 mm and 3.45 mm. The bottom surface crack widths (23 values) produced a higher mean of 4.55 mm, with a standard deviation of 1.84 mm and a margin of error of ± 0.79 mm, placing the confidence interval between 3.76 mm and 5.34 mm.

For the EPS slabs (S2 group), all available bottom surface crack widths were compiled (21 valid values). The mean ($\overline{x}$) was found to be 3.57 mm, with a standard deviation (S.D) of 2.09 mm. The margin of error *(E)* was calculated to be ± 0.95 mm at 0.95 confidence level, indicating a confidence interval for the true mean crack width between 2.62 mm and 4.52 mm.

These results confirm that the control slabs—especially at the bottom surface—sustained greater and more concentrated damage under impact loading. In contrast, the EPS slabs showed reduced average crack widths, though with wider variability due to outlier behavior in specimen S2-3. This variability is attributed to potential inconsistencies in EPS bonding or placement during setup. Future work will incorporate a larger sample size to reduce uncertainty and enable more robust statistical comparison.

Overall, the results suggest that the major failure modes observed in the RC slabs under impact loading included concrete spalling at the impact zone, flexural cracking on the bottom surface, and in some cases, localized punching shear failure. In the control slabs (without EPS), damage was more concentrated and severe near the impact point. The inclusion of the EPS layer helped to redistribute the impact energy over a wider area, reducing the severity of localized damage. In EPS slabs, the failure was more distributed, with reduced spalling and less pronounced cracking patterns. This indicates that the EPS layer contributed to improved energy absorption and helped delay or mitigate critical failure mechanisms. EPS slabs offer better resistance to surface cracking under impact loads compared to control slabs.

**Table 3:** Top cracks values for different points in the specimen.

Specimen type	Specimen ID	1 (mm)	2 (mm)	3 (mm)	4 (mm)	5 (mm)	6 (mm)	7 (mm)	8 (mm)	x̅	S.D	E
Control slab	S1-1	3.54	1.14	4.90	2.1	1.56	2.14	0.75	4.17	2.77	1.53	± 0.68
	S1-2	2.95	3.1	3.95	1.56	3.54	2.12	1.16	1.13			
	S1-3	7.55	2.8	2.1	3.135	3.05	2.56	-	-			
EPS Slab	S2-1	Only hairline cracks										
	S2-2	Only hairline cracks								-	-	-
	S2-3	Only hairline cracks										

**Table 4:** Bottom cracks values for different points in the specimen.

Specimen type	Specimen ID	1 (mm)	2 (mm)	3 (mm)	4 (mm)	5 (mm)	6 (mm)	7 (mm)	8 (mm)	x̅	S.D	E
Control slab	S1-1	2.4	2.8	2.1	2.97	4.1	7.4	3.98	2.97	4.55	1.84	± 0.79
	S1-2	5.51	2.7	5.85	2.96	2.87	3.1	5.4	4.7			
	S1-3	5.8	6.1	9.55	5.1	5.555	6.13	4.6	-			
EPS Slab	S2-1	2.98	3.15	2.3	1.56	1.57	4.55	2.14	2.97	3.57	2.09	± 0.95
	S2-2	2.86	3.9	1.4	3.13	4.7	1.97	2.94	3.26			
	S2-3	9.8	3.55	8.56	4.1	3.5	-	-	-			

**Fig. 9:**
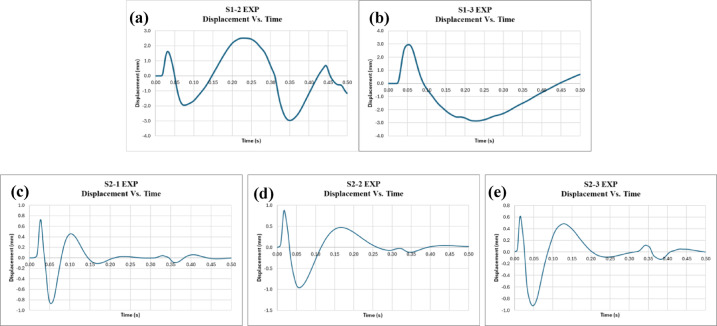
Displacement vs. time for specimens **(a)** S1-2, **(b)** S1-3, **(c)** S2-1, **(d)** S2-2, and **(e)** S2-3.

**Fig. 10:**
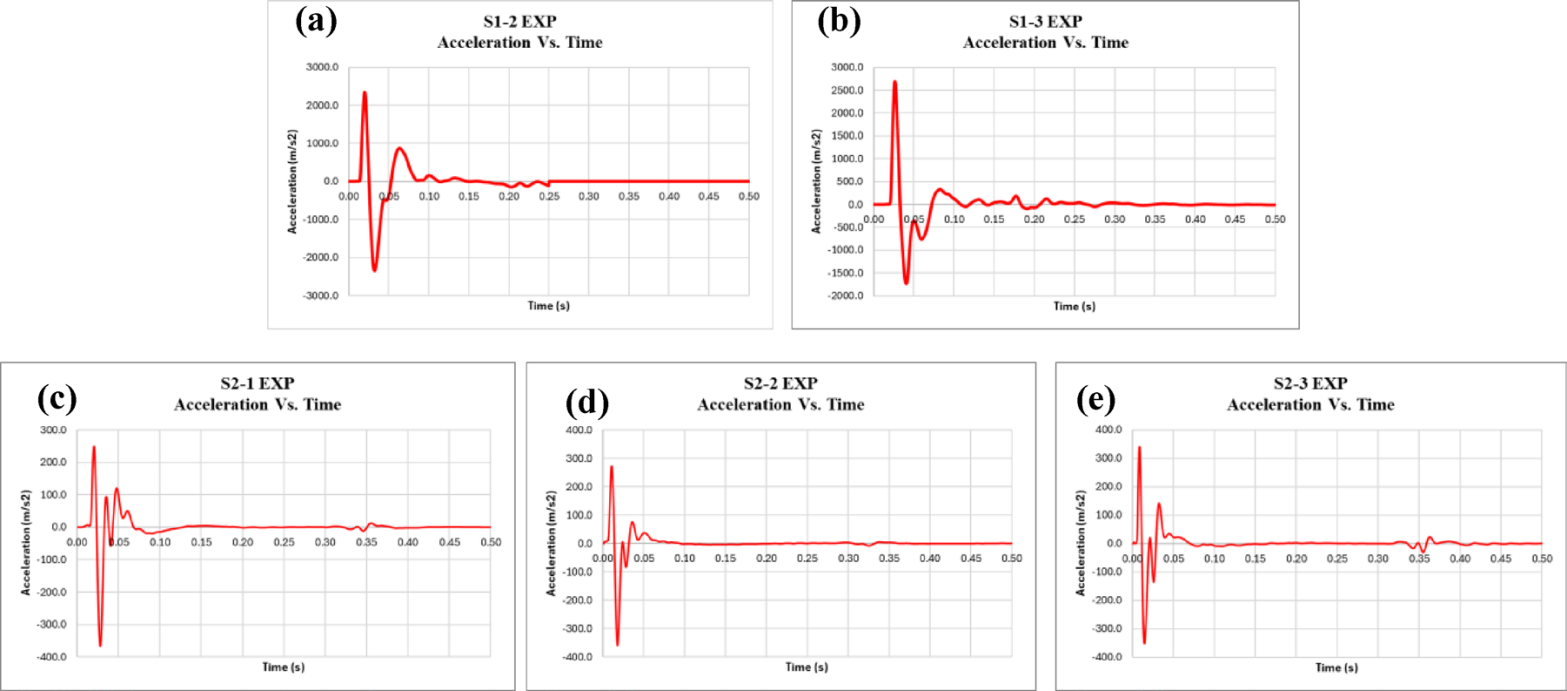
Acceleration vs. time for specimens **(a)** S1-2, **(b)** S1-3, **(c)** S2-1, **(d)** S2-2, and **(e)** S2-3.

**Fig. 11:**
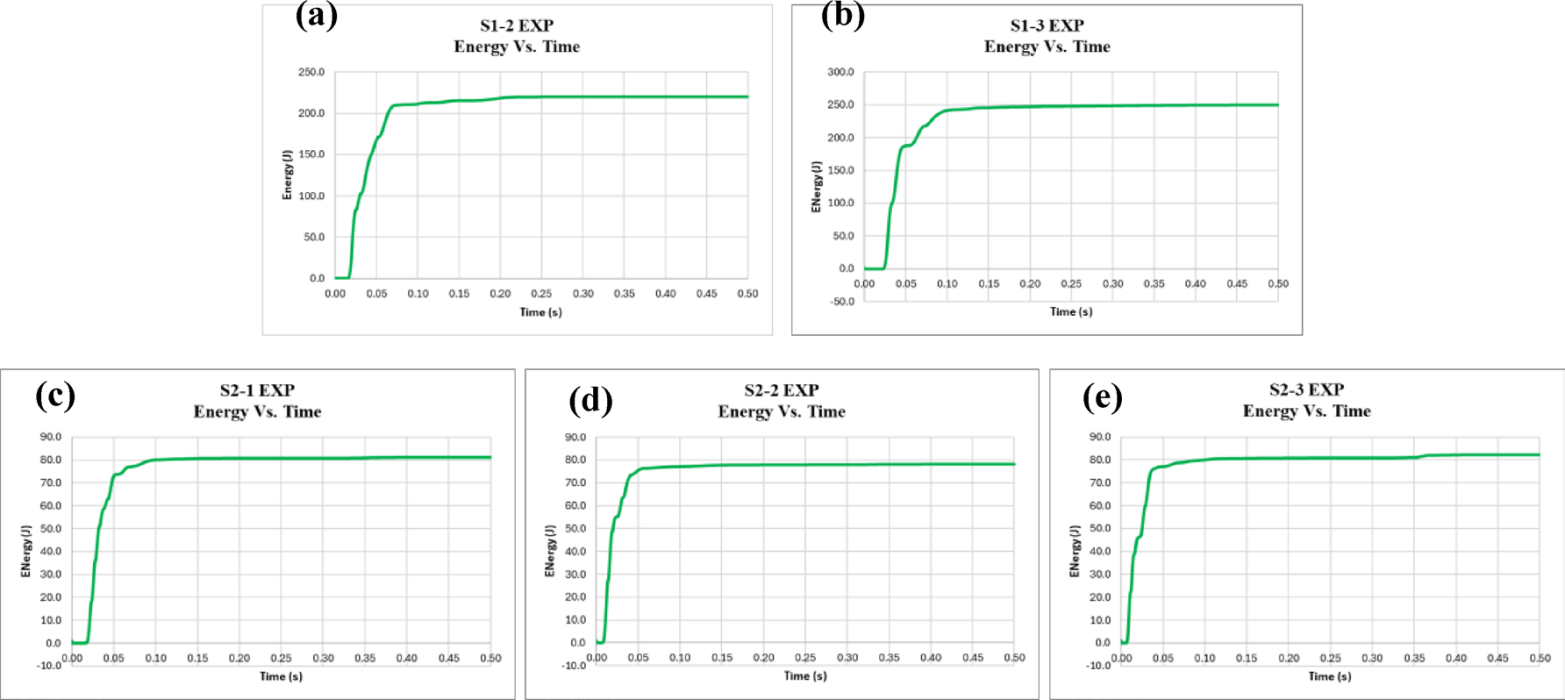
Energy vs. time for specimens **(a)** S1-2, **(b)** S1-3, **(c)** S2-1, **(d)** S2-2, and **(e)** S2-3.

Additionally, the acceleration, velocity, and displacement for all specimens were obtained using an accelerometer placed at the bottom of the slab during the test. The accelerometer measures acceleration, which is then integrated to obtain velocity, and further integrated to obtain displacement. Figures 9, 10 and 11 display the acceleration, velocity, and displacement versus time for all specimens, respectively. Furthermore, the energy dissipated during the impact load is calculated. To calculate the energy dissipated, we start with the given parameters: a mass (m) of 90 kg, acceleration (a) in m/s², and displacement (d) initially given in millimeters. The displacement is first converted from millimeters to meters, with 1 mm equaling 0.001 m. The formula for work done, which represents the energy dissipated, is given by the integral of mass multiplied by acceleration and displacement: Work Done = ∫ (Mass × Acceleration × Displacement). Substituting the known values, we get Work Done = ∫ (90 kg × a m/s² × Disp. (m)).

The results of the impact load test on both the control and EPS slabs, as summarized in Table 4, show distinct differences in performance metrics. For the control slabs, the average maximum acceleration recorded was 2520.38 m/s², with individual values ranging from 2350.34 m/s² to 2690.43 m/s². The average maximum displacement was 2.96 mm, with individual values between 2.95 mm and 2.97 mm. The energy dissipated by the control slabs averaged 234.92 Joules, with a range from 219.93 Joules to 249.90 Joules. In comparison, the EPS slabs showed a lower average maximum acceleration of 359.09 m/s², with values ranging from 351.40 m/s² to 366.79 m/s². The average maximum displacement for the EPS slabs was 0.92 mm, with values between 0.87 mm and 0.96 mm. Notably, the energy dissipated by the EPS slabs was significantly lower, averaging 80.50 Joules, with individual values ranging from 78.15 Joules to 82.21 Joules.

In comparison, the experimental results for specimens S1 and S2, as shown in Table 5, further emphasize the difference between the two types of slabs. For displacement, the control slabs (S1) had a maximum displacement of 2.96 mm, while the EPS slabs (S2) exhibited a much lower maximum displacement of 0.92 mm, resulting in a 69% reduction. This could be attributed to the idea that less structural deformation occurred in the EPS slabs, not because they absorbed less energy overall, but because the EPS layer shielded the concrete from the full intensity of the impact. With reduced internal stresses and crack propagation, the concrete experiences less movement and distortion. Regarding acceleration, the control slabs had a maximum acceleration of 2520.38 m/s², while the EPS slabs showed a significantly lower value of 359.09 m/s², which represents an 86% reduction. The explanation for this reduction is likely to be a direct consequence of the EPS layer’s cushioning effect, which absorbs and attenuates the initial shock from the impact before it reaches the RC slab. EPS deforms under load, spreading the impact energy over a longer duration and reducing the rate of force transmission. Since acceleration is highly sensitive to how quickly a force is applied, this delay and distribution in force transfer significantly lowers peak acceleration values. As for energy dissipation, the control slabs dissipated 234.92 Joules on average, while the EPS slabs absorbed much less energy, with a value of 80.5 Joules. This leads to a 66% reduction, underscoring the much greater energy dissipation in the control slabs compared to the EPS slabs.

The observation that control slabs (without EPS) dissipated more energy than the EPS slabs may initially seem counterintuitive, as the EPS layer is intended to enhance impact performance. However, this finding can be explained by examining the fundamental mechanisms through which energy dissipation occurs during impact. In the control slabs, the entire impact energy is directly transferred to the reinforced concrete section, resulting in more extensive material damage such as surface cracking, spalling, internal microcracking, and in some cases, crushing of the concrete at the impact zone. These forms of structural degradation are energy-intensive processes, and thus the energy dissipation recorded for these specimens appears higher.

Conversely, in the EPS slabs, the EPS layer acts as an energy-absorbing buffer between the impactor and the RC slab. Due to its low stiffness and deformability, the EPS layer deforms under impact, absorbing a portion of the kinetic energy through localized compression and mitigating the force transmitted to the concrete surface. This cushioning effect results in a significant reduction in the stress and strain levels experienced by the concrete, thereby reducing the extent of physical damage and the associated energy dissipation mechanisms. In essence, while the total energy imparted by the impact is the same, the distribution and mode of dissipation differ — with the EPS layer absorbing part of the energy through elastic or quasi-elastic deformation, and the RC slab sustaining lower levels of damage. This ultimately results in lower overall energy dissipation being recorded from the slab itself. Therefore, the lower energy dissipation in the EPS slabs is not indicative of poorer impact performance; on the contrary, it demonstrates a more efficient and protective response, where less damage to the structural component is achieved through the strategic inclusion of a sacrificial and energy-absorbing layer.

These findings confirm that the EPS layer did not reduce impact performance but rather altered how and where energy was absorbed. In control slabs, more energy was dissipated within the concrete through cracking and failure mechanisms—processes that inherently involve large deformations and energy loss. In contrast, the EPS slabs allowed a portion of the energy to be absorbed by localized compression within the EPS, preserving the integrity of the RC slab and thus requiring less energy dissipation through damage.

**Table 4:** acceleration, velocity, displacement, and energy dissipated for all experimental specimens.

Specimen type	Specimen ID	Max displacement (mm)	Max Acceleration (m/s2)	Energy (Joules)
Control Slab	S1-2	2.97	2350.34	219.93
	S1-3	2.95	2690.43	249.90
	**Avg.**	**2.96**	**2520.38**	**234.92**
EPS Slab	S2-1	0.87	366.79	81.14
	S2-2	0.96	359.07	78.15
	S2-3	0.92	351.40	82.21
	**Avg.**	**0.92**	**359.09**	**80.50**

**Table 5:** Comparison between results of experimental specimens.

Exp Specimen	Max displacement (mm)	Max Acceleration (m/s2)	Energy (Joules)
S1	2.96	2520.38	234.92
S2	0.92	359.09	80.5
Reduction %	69	86	66

## Finite element model

### FEM boundary conditions and loading

**Fig. 12:**
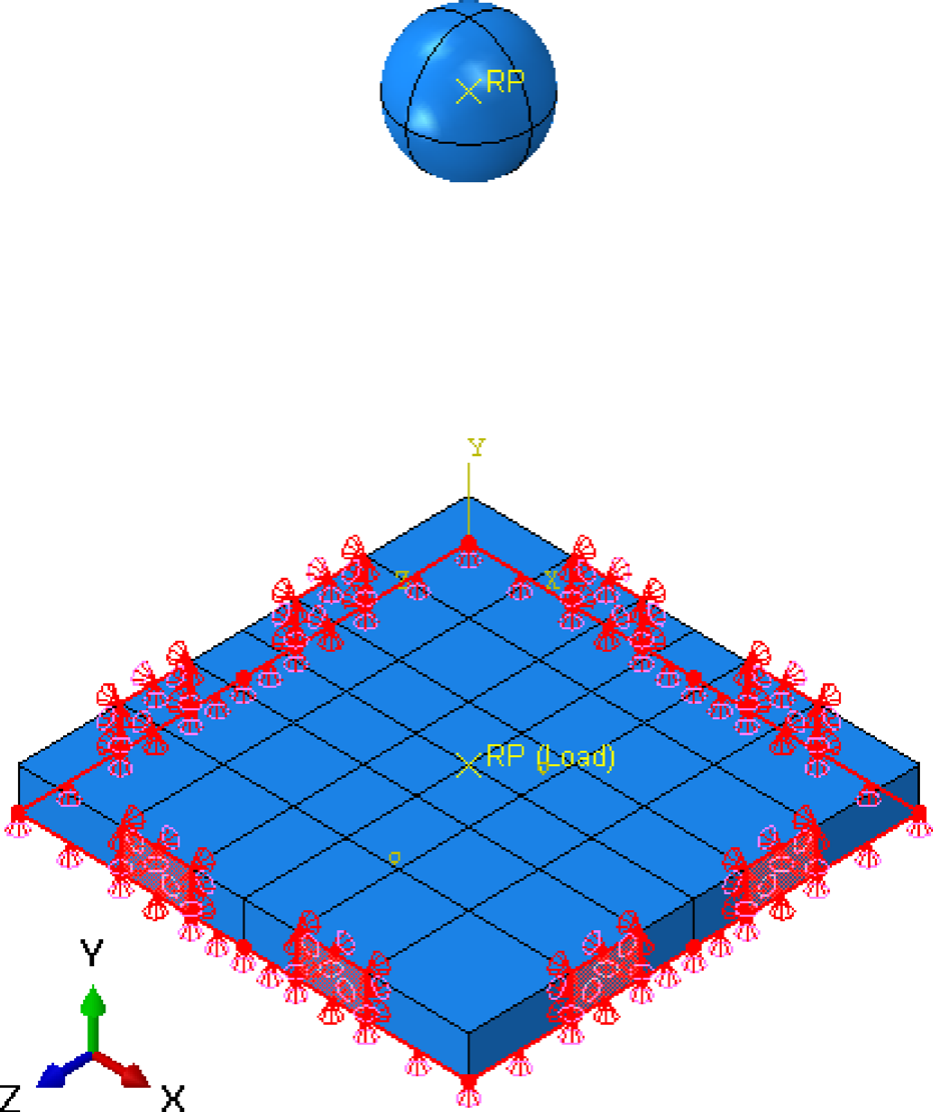
Boundary condition for numerical slab model.

In the numerical model using ABAQUS^11^, the boundary conditions and loading are crucial for accurately replicating the experimental setup. To simulate the experimental conditions, the bottom edge of the slab is restrained in the Y-direction (U2 = 0), while the side edges of the slab are restrained in horizontal direction (X or Z-direction) and in Y-direction to simulate the effect of the clamps. This restrains the translational degrees of freedom (U2 = U3/or U1 = 0) on the side parts of the slab, ensuring the model accurately reflects the full fixation as in the experimental constraints, as shown in Fig. 12.

The impact load is simulated by dropping a steel ball onto the slab. To achieve this, a dynamic explicit step is defined in ABAQUS, allowing for the capture of the transient response of the slab under impact load. The steel ball, modeled as a discrete rigid body, is assigned an initial velocity based on the experimental setup. Gravity is included in the simulation to ensure realistic loading conditions. The initial velocity of the steel ball is derived from the equation $\:V=\:\sqrt{2gh}$, where v is velocity, g is the acceleration due to gravity, and h is the height from which the ball is dropped. This setup replicates the impact scenario and provides detailed insights into the slab’s response over time.

### FEM of control model

**Fig. 13:**
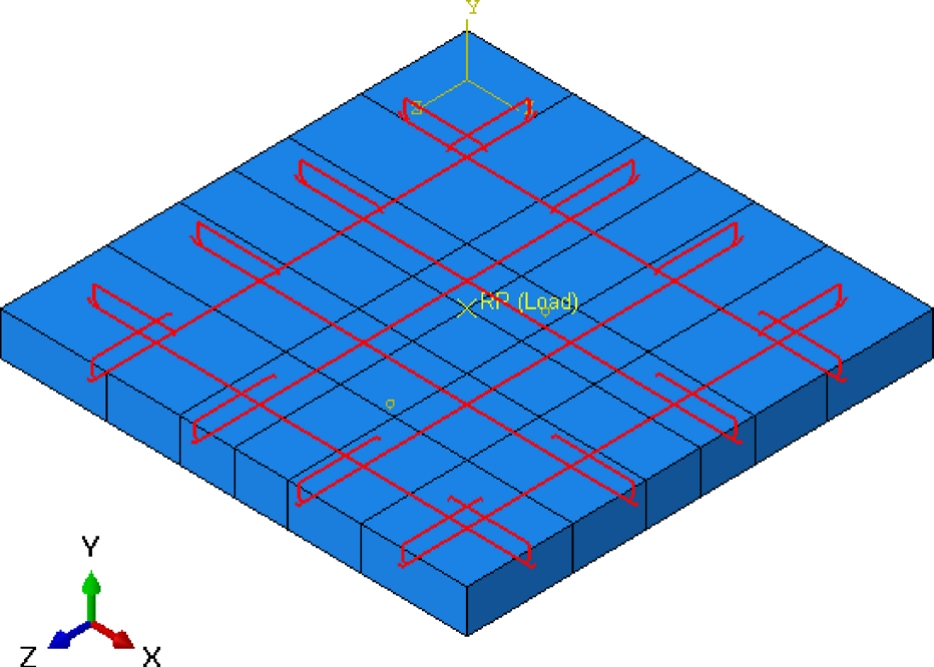
Numerical model of control slab.

The FEM model for the control slab, which does not incorporate expanded polystyrene (EPS), is created in ABAQUS^11^ as a 3D solid model as shown in Fig. 13. The slab dimensions are defined according to the experimental setup, typically measuring 900 mm x 900 mm x 80 mm. The material properties for the concrete are specified, including a density of 2500 kg/m^3^, a Young’s modulus of 23186.7 MPa, and a Poisson’s ratio of 0.2. These properties ensure that the concrete’s behavior under load is accurately simulated.

The steel reinforcement within the slab is modeled using wire elements. The reinforcement is divided into bottom and top layers, with main and secondary bars specified. The material properties of the steel are defined, with a Young’s modulus of 200,000 MPa and a Poisson’s ratio of 0.3. These reinforcement bars are embedded within the concrete slab using the embedded region constraint in Abaqus, ensuring proper interaction between the concrete and steel.

**Fig. 14:**
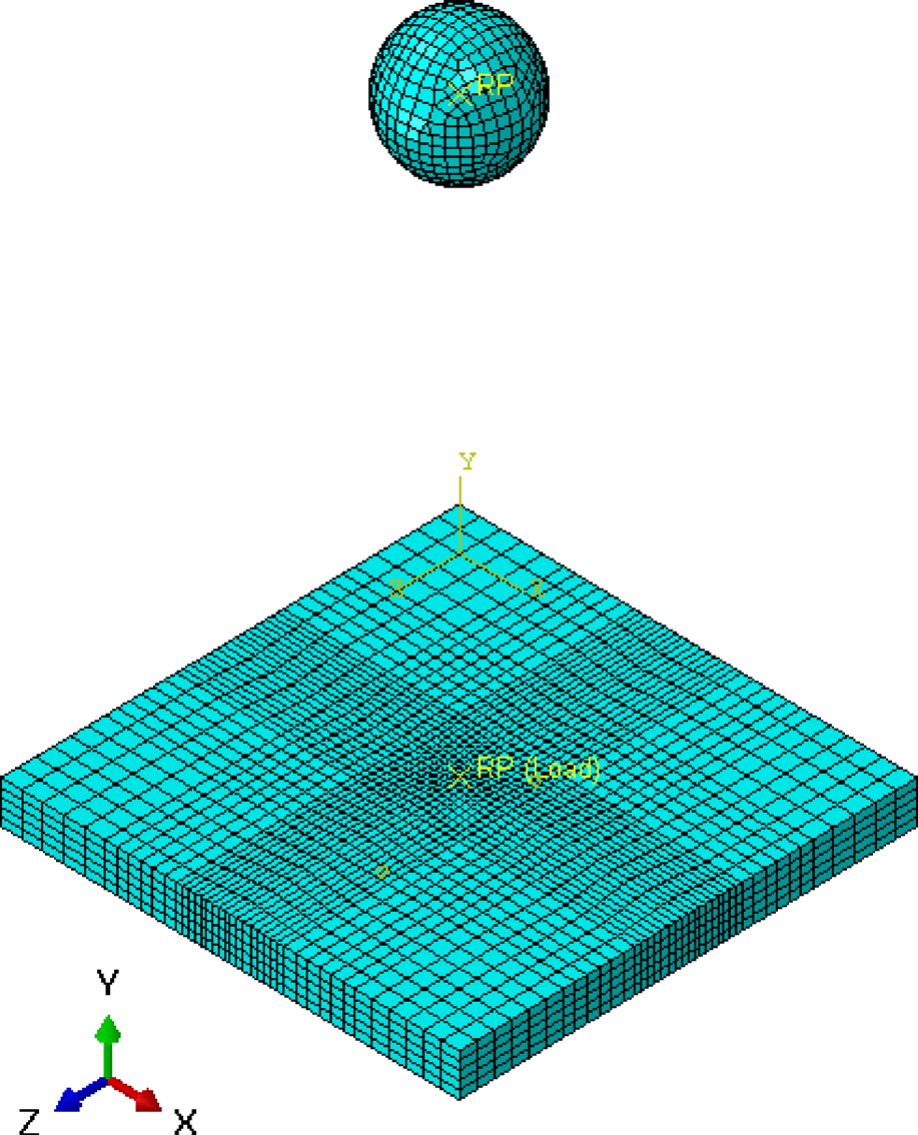
Mesh modelling of control slab.

The mesh for the control slab is generated with a global size of approximately 50 mm for the slab and 10 mm for the reinforcement bars, as shown in Fig. 14. This meshing strategy ensures a balance between accuracy and computational efficiency, capturing critical stress and strain distributions within the slab.

### FEM of EPS model

**Fig. 15:**
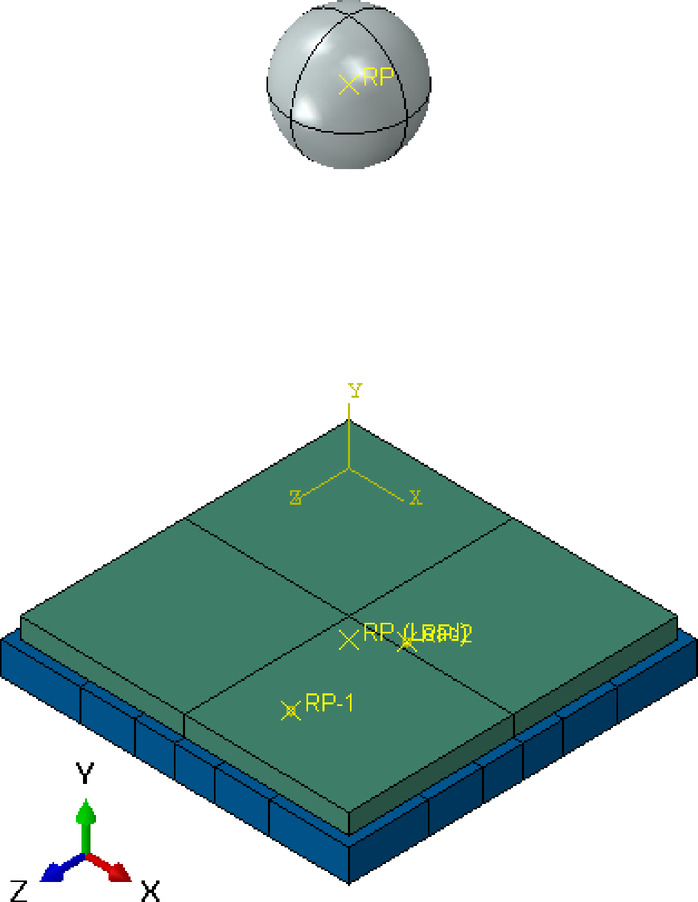
numerical model of EPS slab.

The FEM model for the EPS slab follows a similar approach but includes an additional layer of expanded polystyrene within the slab as shown in Fig. 15. The EPS layer is modeled as a separate part within the slab assembly, with its position and thickness accurately represented. The material properties of EPS are defined, considering its lower density and different mechanical properties compared to concrete. Typical properties include a density of 100 kg/m^3^ and a lower Young’s modulus, reflecting the material’s compressibility and energy absorption characteristics.

The interaction between the EPS and concrete layers is defined using appropriate contact properties to simulate the real behavior under impact loading. This includes specifying tangential and normal contact behavior to accurately capture the interface response. In the normal direction, a hard contact formulation was used, allowing for separation after contact, thereby capturing the potential for partial detachment at the interface during impact. In the tangential direction, a penalty friction formulation was applied to simulate the potential for limited sliding between the EPS and concrete surfaces. Based on experimental findings reported in the literature, a friction coefficient of 0.4 was assigned to the EPS–concrete interface, consistent with values used in similar contact simulations^15^. The reinforcement in the EPS slab is modeled similarly to the control slab, using wire elements for the steel bars with the same material properties.

To further enhance the accuracy of the contact interaction, the mesh for the EPS slab is generated with a focus on the interface between the EPS and concrete layers. A finer mesh is applied at the interface to ensure the transition is accurately captured. This meshing strategy helps in analyzing the impact of the EPS layer on the overall performance of the slab under dynamic loading conditions.

### Results of FEM model

**Fig. 16:**
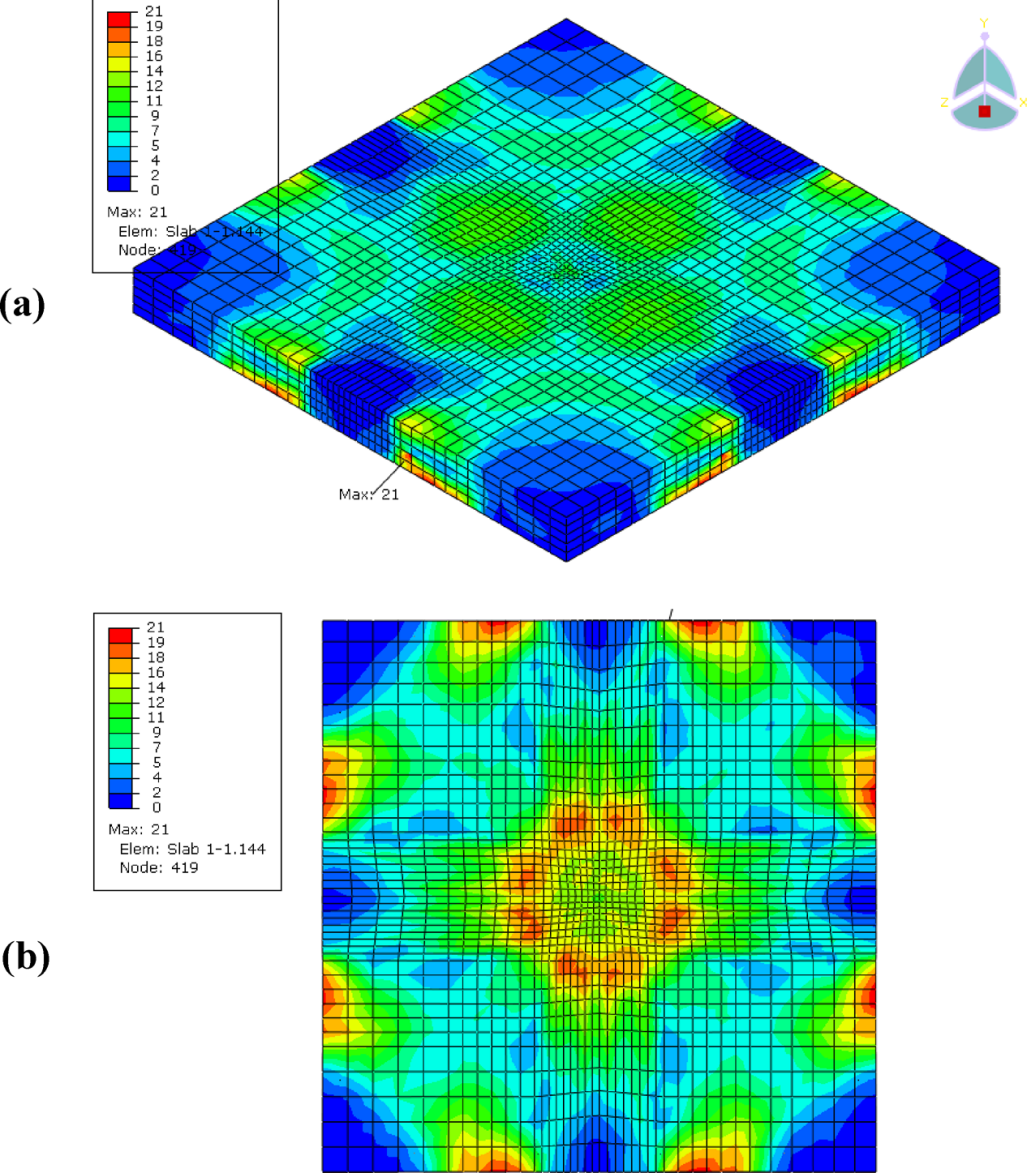
Stress distribution and deformation patterns in FE model control slab **(a)** 3D view and **(b)** bottom of slab.

**Fig. 17:**
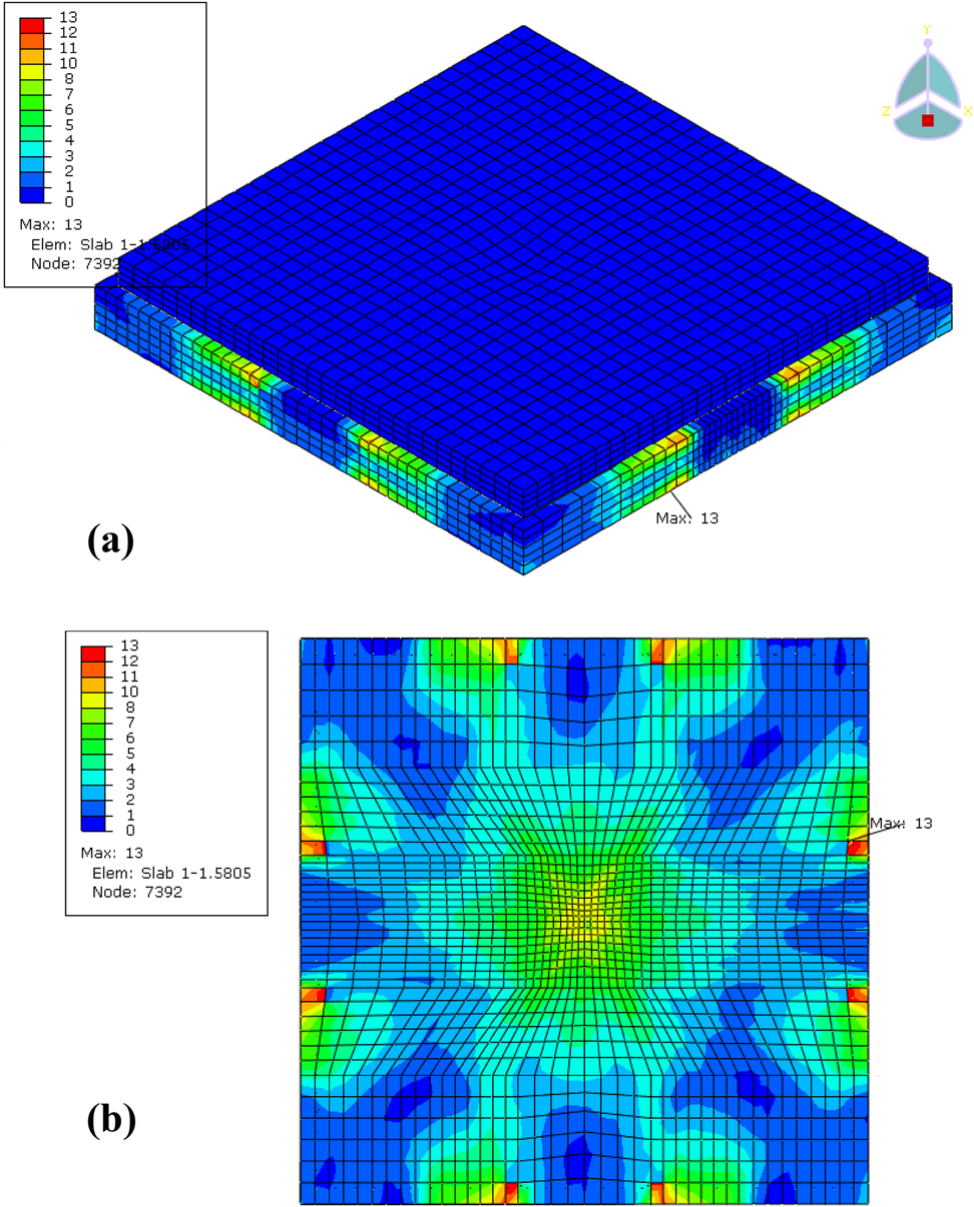
Stress distribution and deformation patterns FE model EPS slab **(a)** 3D view and **(b)** bottom of slab.

The analysis results showing the stress distribution and deformation pattern of the control slab (reinforced concrete only) and the EPS slab (reinforced concrete with an EPS layer) under impact loading, is shown in Figs. 16 and 17, respectively. The control slab exhibited higher stress concentrations, with a maximum stress of 21 MPa, primarily located at the corners and central zones of the slab. This indicates that the rigid nature of the reinforced concrete effectively resists deformation but results in localized stress intensities, particularly in critical areas. The rigidity of the control slab enables it to absorb and distribute energy through the concrete, but this comes at the cost of creating stress hotspots, which may lead to localized damage under high-impact conditions.

In contrast, the EPS slab demonstrated significantly lower stress levels, with a maximum stress of 13 MPa, reflecting the effectiveness of the EPS layer in mitigating stress transfer to the reinforced concrete layer. The stress in the EPS slab was more localized along the edges and less intense in the central areas, indicating the cushioning effect of the EPS layer. By introducing flexibility, the EPS layer absorbs and dissipates a portion of the impact energy, reducing the overall stress and protecting the underlying concrete from severe force transmission.

Comparatively, the control slab’s higher stiffness and rigidity make it more suitable for scenarios requiring resistance to deformation and concentrated energy absorption. However, the EPS slab’s enhanced flexibility and ability to distribute forces make it ideal for impact mitigation applications. Overall, the inclusion of the EPS layer significantly reduces stress concentrations and provides better protection for the structure under dynamic loading conditions.

**Fig. 18:**
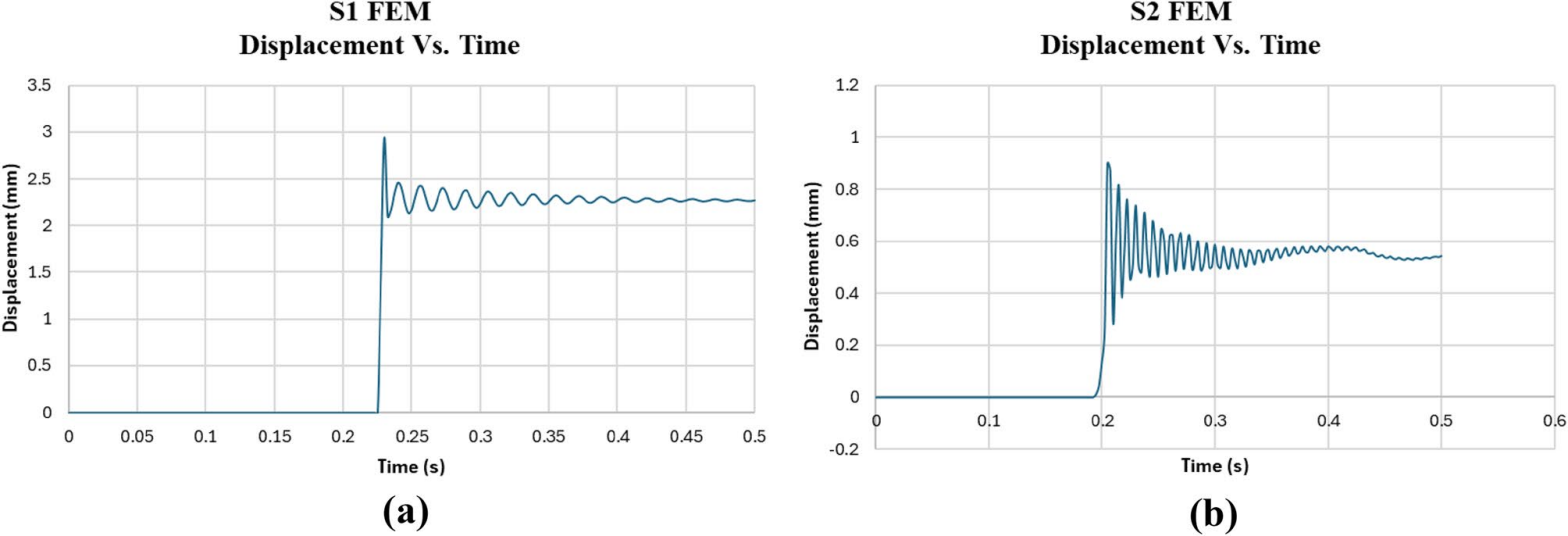
Displacement Vs. Time for **(a)** numerical control model and **(b)** numerical EPS model.

**Fig. 19:**
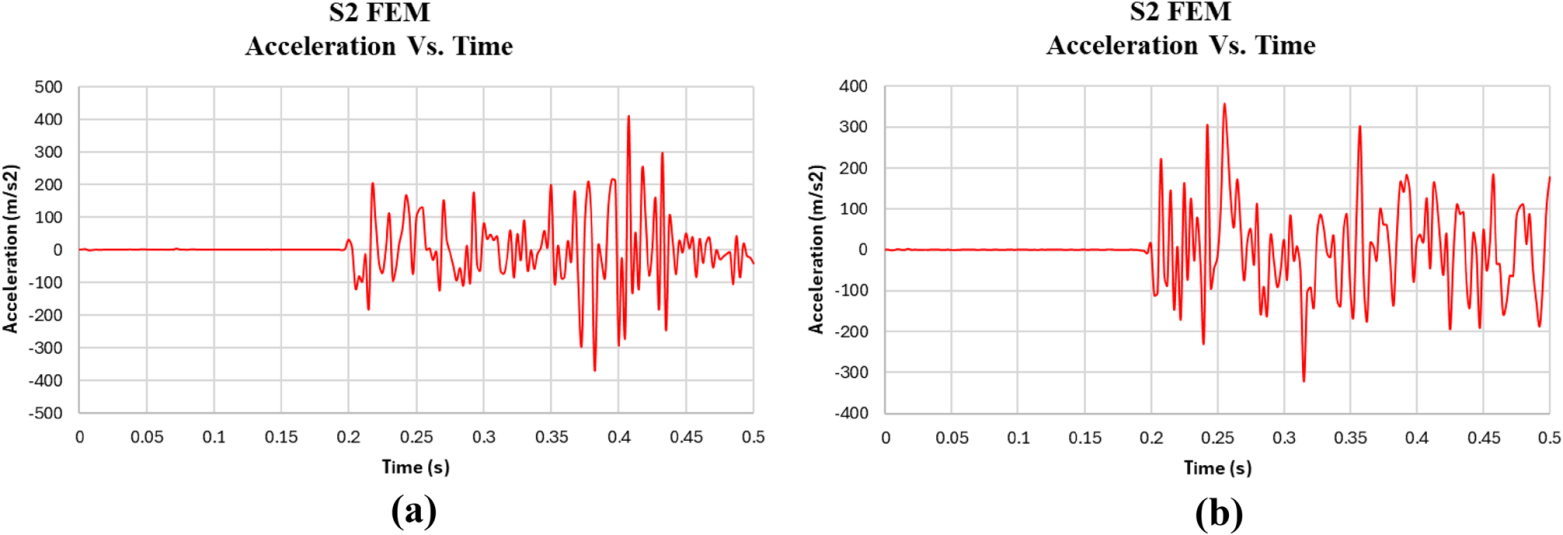
Acceleration Vs. Time for **(a)** numerical control model and **(b)** numerical EPS model.

**Fig. 20:**
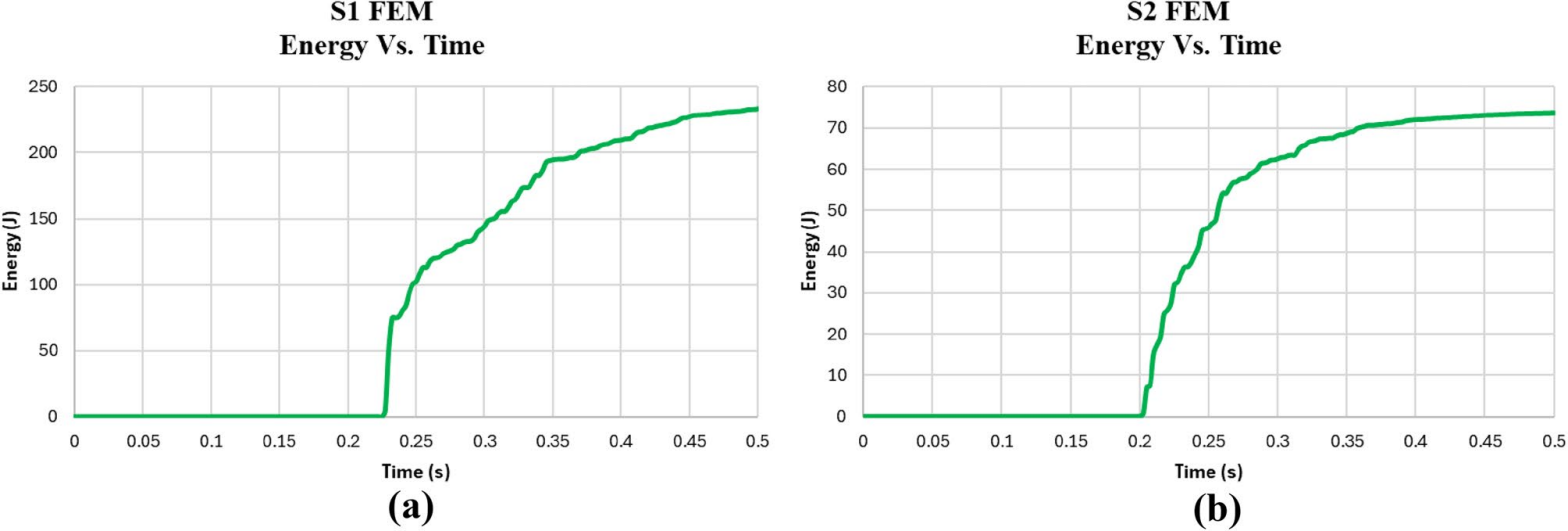
Energy Vs. Time for **(a)** numerical control model and **(b)** numerical EPS model.

The results of the FEM analysis for the control (S1) and EPS (S2) models are discussed in detail in terms of displacement, acceleration, and energy absorbed, as shown in Figs. 18, 19 and 20. The results indicate that the FEM Control model (S1) experienced a significantly higher peak acceleration (2444 m/s²) compared to the FEM EPS model (S2), which had a peak acceleration of 349.9 m/s².

Maximum acceleration is a critical measure of how quickly the slab decelerates upon impact, offering insights into the structural dynamics and the effectiveness of the materials in absorbing kinetic energy. The substantially lower acceleration observed in the FEM EPS model suggests that the EPS layer effectively cushions the impact by absorbing and distributing the force over a longer duration, reducing the sudden deceleration experienced by the slab. In contrast, the control slab (S1), composed entirely of reinforced concrete, exhibits a rigid response, resulting in a much sharper and more sudden deceleration.

The results also show that the FEM control slab (S1) underwent a larger maximum displacement (2.93 mm) compared to the FEM EPS slab (S2), which had a maximum displacement of 0.90 mm. This result is somewhat counterintuitive but can be explained by the differences in material behavior under impact. The EPS layer in the S2 model adds stiffness by mitigating the transmission of impact forces, thereby reducing the overall deformation. Meanwhile, the Control slab’s rigidity and lack of a cushioning layer led to a greater concentration of stress at the point of impact, resulting in a larger displacement.

In terms of energy absorption, the FEM Control slab absorbed significantly more energy (233 Joules) than the FEM EPS slab (78.1 Joules). This highlights the inherent energy dissipation capacity of reinforced concrete, which can absorb and dissipate energy through mechanisms like microcracking and plastic deformation. The absence of an EPS layer in the Control model allows the slab to resist deformation and absorb more energy directly. Conversely, the FEM EPS model, while effective at cushioning the impact, does not absorb as much energy, as the EPS layer primarily acts as a shock absorber rather than a material with high energy dissipation capacity. The reduced energy absorption in the FEM EPS slab indicates that the impact forces are mitigated by allowing lower acceleration and displacement but at the cost of reduced energy dissipation.

Overall, the FEM analysis underscores the distinct advantages of using an EPS layer in reducing both displacement and peak acceleration, demonstrating its potential for applications requiring impact mitigation. However, the lower energy absorption in the FEM EPS model may limit its effectiveness in scenarios demanding higher energy dissipation.

The impact load test results, as shown in Table 6, highlight the efficiency of the EPS slab (S2) in reducing impact energy compared to the control slab (S1). The control slab had a max displacement of 2.93 mm and max acceleration of 2444 m/s², both of which were significantly higher than the EPS slab, which had a max displacement of 0.90 mm and max acceleration of 349.9 m/s². In terms of calculated energy, the control slab absorbed 234.92 Joules, while the EPS slab absorbed only 78.1 Joules, indicating a 97.9% reduction in energy for the EPS slab. This demonstrates that the EPS slab is highly efficient at reducing the energy of impact, resulting in lower deformation and acceleration. Therefore, the EPS slab is more effective in minimizing the impact energy, while the control slab absorbs more energy but experiences greater displacement and acceleration.

**Table 6:** Comparison between the results of FE models.

FE Model	Max displacement (mm)	Max Acceleration (m/s2)	Energy (Joules)
S1	2.93	2444	234.92
S2	0.90	349.9	78.1
Reduction %	69	85	67

### FEM verification

The results shown in Table 7, compare experimental data (Exp) and finite element model (FEM) simulations performed using ABAQUS for both control slabs and EPS slabs. The displacement, acceleration, and energy values from the experimental tests are compared with those from the FE model for each specimen.

For the control slab (S1), the experimental displacement was 2.97 mm (S1-2) and 2.95 mm (S1-3), with an average of 2.96 mm, while the FE model yielded a displacement of 2.93 mm. The maximum acceleration for the experimental results was 2350.34 m/s² (S1-2) and 2690.43 m/s² (S1-3), with an average of 2520.38 m/s², whereas the FE model showed a value of 2444.07 m/s². The calculated energy for the experimental model was 219.93 MJ (S1-2) and 249.90 MJ (S1-3), with an average of 234.92 MJ, compared to 233.64 MJ for the FE model. The variance in displacement and acceleration for the control slab was relatively small, with 0.98% and 3.07%, respectively.

For the EPS slab (S2), the experimental displacement ranged from 0.87 mm (S2-1) to 0.96 mm (S2-2), with an average of 0.92 mm, while the FE model predicted a displacement of 0.90 mm. The maximum acceleration for the experimental model was 366.79 m/s² (S2-1) and 359.07 m/s² (S2-2), with an average of 359.09 m/s², compared to 349.85 m/s² from the FE model. The energy dissipated in the experiments ranged from 78.15 MJ (S2-2) to 82.21 MJ (S2-3), with an average of 80.50 MJ, while the FE model predicted 78.06 MJ, showing a 3.07% variance.

The finite element model (FEM) using ABAQUS was verified against the experimental results with minimal variance in displacement, acceleration, and energy. The FE model is found to be suitable and provides accurate results, confirming its reliability for simulating the behavior of both control and EPS slabs under impact loads. The close agreement between the experimental and FEM results shows that the model can reflect the actual physical response of the slabs, making it an effective and suitable method for structural analysis in this context and for future parametric studies.

**Table 7:** Comparison between experimental and FE model results.

Specimen type	Type	Specimen ID	Displacement (mm)	Acceleration (m/s2)	Energy (J)
**Control Slab**	EXP	S1-2	2.97	2350.34	219.93
		S1-3	2.95	2690.43	249.90
		Avg.	**2.96**	**2520.38**	**234.92**
	FEM	S1	**2.93**	**2444.07**	**233.64**
	VAR. %		**0.98**	**3.07**	**0.55**
**EPS Slab**	EXP	S2-1	0.87	366.79	81.14
		S2-2	0.96	359.07	78.15
		S2-3	0.92	351.40	82.21
		Avg.	**0.92**	**359.09**	**80.50**
	FEM	S2	**0.90**	**349.85**	**78.06**
	VAR. %		**1.77**	**2.61**	**3.07**

## Conclusion

Based on the work presented, test setup, loading conditions, boundary conditions and materials used, the following points were concluded:

- The EPS layer functioned as a cushioning medium that helped reduce the severity of the impact force transmitted to the slab. This was evident in the acceleration and displacement responses observed in the slabs with EPS compared to the control slabs. The EPS absorbed part of the impact energy, thereby reducing the damage and delaying crack propagation without compromising the inherent stiffness or strength of the RC slab.
- The experimental results showed that the EPS slab reduced displacement by 69%, acceleration by 86%, and energy dissipation by 66% when compared to the control slabs, highlighting its superior performance in minimizing impact effects.
- Similarly, the finite element (FE) model results confirmed that the EPS slab reduced displacement by 69%, acceleration by 85%, and energy dissipation by 67%, further validating the material’s effectiveness in energy absorption and impact mitigation.
- The results indicate that the FE model aligns well with the experimental findings, with discrepancies in displacement, acceleration, and energy dissipation showing variances of no more than 1.77%, 3.07%, and 3.07%, respectively. This minimal variance affirms the accuracy and reliability of the FE model for simulating EPS slab behavior under impact loads which makes it very powerful for further future studies.
- The inherent physical properties of EPS, including its low density, compressibility, and energy-dissipating capabilities, make it an ideal material for absorbing impact loads. EPS reduces the transmitted force and protects the materials beneath it from damage, offering an efficient and cost-effective solution for impact resistance in structural applications.

To broaden the applicability of this research, comprehensive parametric study is planned as part of future work. This will involve systematically varying concrete properties, steel reinforcement, load intensity and EPS parameters—including density, thickness, and mechanical characteristics—to better understand their influence on the impact performance of reinforced concrete slabs. Such work aims to establish more generalized relationships and design guidelines that can inform practical applications, such as those involving different EPS grades or configurations used in insulating concrete form (ICF) systems or protective overlays.

## Data Availability

The datasets generated and/or analyzed during the current study are not publicly available as they are part of ongoing research. However, they are available from the corresponding author upon reasonable request.
